# Multiple peripheral typical carcinoid tumors of the lung: associated with sclerosing hemangiomas

**DOI:** 10.1186/1746-1596-8-97

**Published:** 2013-06-17

**Authors:** Young Kim, Yoo-Duk Choi, Beum Jin Kim, In-Jae Oh, Sang-Yun Song, Jong-Hee Nam, Chang-Soo Park

**Affiliations:** 1 Department of Pathology, Chonnam National University Medical School, 5 Hak-dong, Dong-gu, 501-746 Gwangju, Republic of Korea; 2 Tissue Pathology and Diagnostic Oncology, Royal Prince Alfred Hospital, Sydney, Australia; 3 Department of Internal Medicine, Chonnam National University Medical School, Gwangju, Republic of Korea; 4 Department of Thoracic and Cardiovascular Surgery, Chonnam National University Medical School, Gwangju, Republic of Korea; 5 Brain Korea 21 project, Center for Biomedical Human Resources at Chonnam National University, Gwangju, Republic of Korea

**Keywords:** Carcinoid tumors, Sclerosing hemangiomas, Lung

## Abstract

**Abstract:**

This study presents a first case of multiple peripheral typical carcinoid tumors associated with sclerosing hemangiomas in the lung. A 52-year-old male presented with incidentally detected multiple pulmonary nodules on a simple chest X-ray during routine health check-up. A computed tomography (CT) scan of the chest showed multiple nodular lesions in the middle and lower lobes of the right lung. These were initially suspected as inflammatory lesions due to miliary tuberculosis. However, possibility of malignancy could not be excluded and right lower lobe lobectomy was performed. Histopathologically, some nodules including two largest nodules were composed of small round to spindle shaped cells with fine chromatin pattern, whereas the rest of the sclerotic nodules were composed of two epithelial cell types- surface cells and round cells. The final diagnosis of this case was multiple peripheral typical carcinoid tumors associated with sclerosing hemangiomas of the lung. For past three years of post-surgery follow up period, no new lesions or changes in the right middle lobe have been identified.

**Virtual Slides:**

The virtual slide(s) for this article can be found here: http://www.diagnosticpathology.diagnomx.eu/vs/1511610609725790.

## Background

Pulmonary sclerosing hemangioma (PSH) is an uncommon lung tumor, characterized as alveolar pneumocytoma. Since the first report in 1956 by Liebow and Hubbel [1], PSH has been considered to be a benign pulmonary tumor occurring predominantly in females that usually presents as peripheral solitary lesions [2]. Despite several studies of PSH, its clinical behavior and histogenesis still remains ambiguous. However, the histologic characteristic of PSH is well known to show papillary, sclerotic, solid, and hemorrhagic patterns with two cell types [2]. Multiple studies report cases about PSH with unconventional histological morphologies: PSH presenting in multiple nodules of atypical adenomatous hyperplasia, PSH containing adenocarcinoma-like portion, coexistence of PSH with primary adenocarcinoma, and PSH with metastatic hereditary non-polyposis colorectal cancer [3-6]. However, there has never been a publication about multiple typical carcinoids found simultaneously with sclerosing hemangiomas within the same lobe of the lung. This study reports a first case of multiple peripheral typical carcinoids associated with sclerosing hemangiomas within the same lobe of the right lung.

### Case presentation

**Figure 1:**
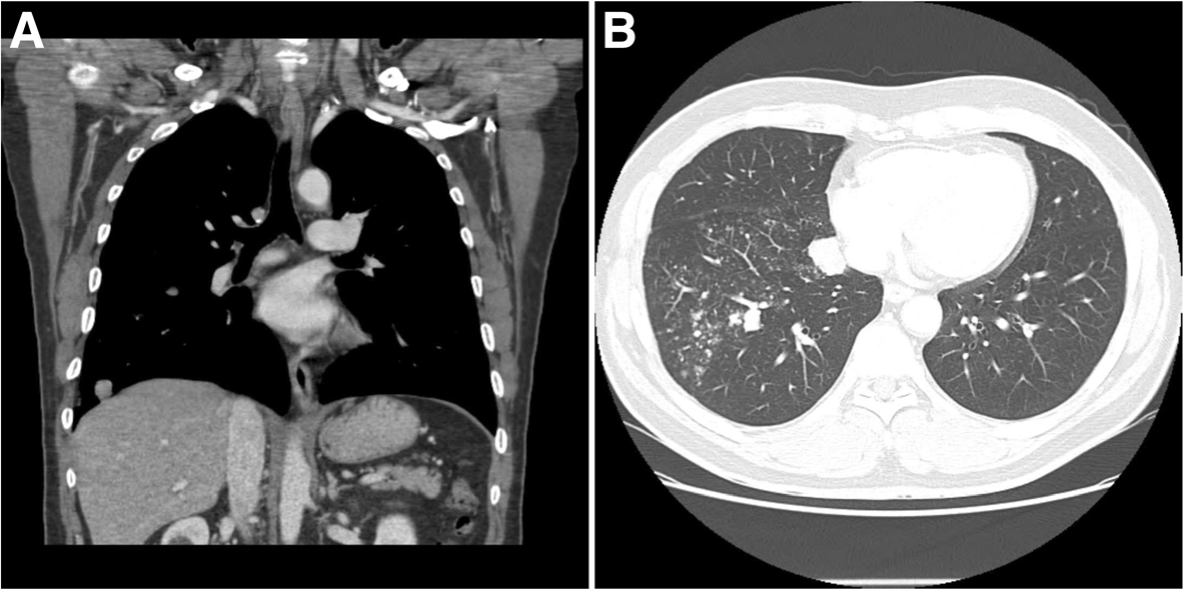
**Preoperative chest computed tomography.** (**A**, **B**) CT shows numerous nodules with some calcifications in the right middle and lower lobes.

A 52-year-old man with 2 years history of right lung lesion, which was incidentally detected on a chest X-ray, visited our hospital for further investigation and follow up. Patient had a past history of 40 pack years of cigarette smoking, frequent alcohol drinking, and pulmonary tuberculosis as a child. Patient was asymptomatic and there were no abnormal findings on physical examination, laboratory investigation and pulmonary function test. Chest computed tomography (CT) of the lung showed diffuse reticulonodular densities, tiny calcified nodules, and several larger confluent nodules in the right middle and lower lobes (Figure 1). These findings were equivalent to previous results from 2 years ago. Tuberculosis culture of the sputum and the bronchial aspiration fluids were performed, but there was no organism growth observed and both were negative for TB-PCR. Stable miliary tuberculosis was the initial clinical impression for this case. However, since possibility of malignancy could not be excluded, video-associated right lower lobe lobectomy was performed for a definitive diagnosis.

**Figure 2:**
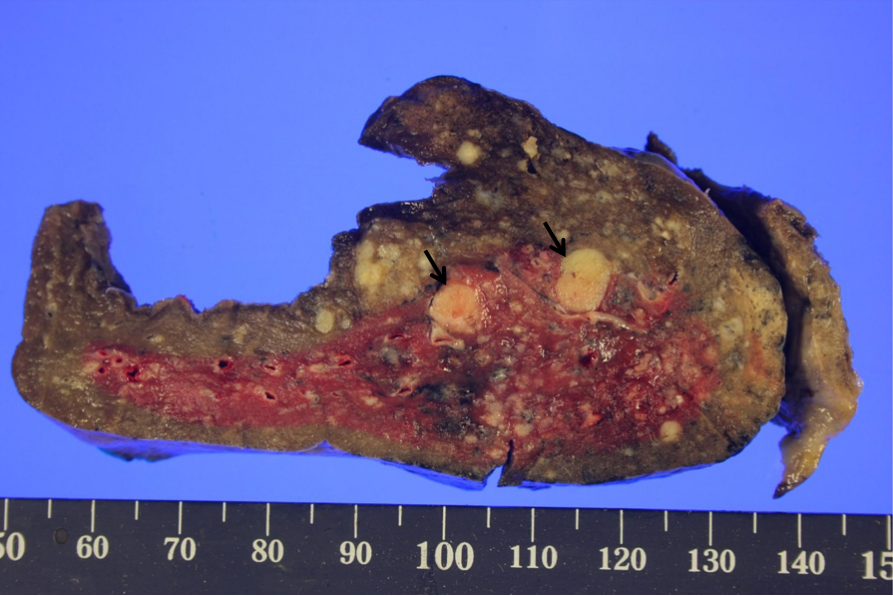
**Postoperative macroscopic examination of the lung.** On gross examination, the tumors were white to pink in color, variable in size, with some calcifications and hemorrhages. They were well circumscribed round shaped tumors without fibrous capsule. The arrows indicate carcinoid tumors.

Gross examination of the right lower lobe showed multiple well-circumscribed gray-red and white nodules inside the lung parenchyma, interlobar fissure, and also on the visceral pleura (Figure 2). These nodules were not encapsulated and were variable in size ranging in diameter from 5 mm to 26 mm. The largest nodule of these was located inside the lung parenchyma, measuring 26 × 20 mm in size.

**Figure 3:**
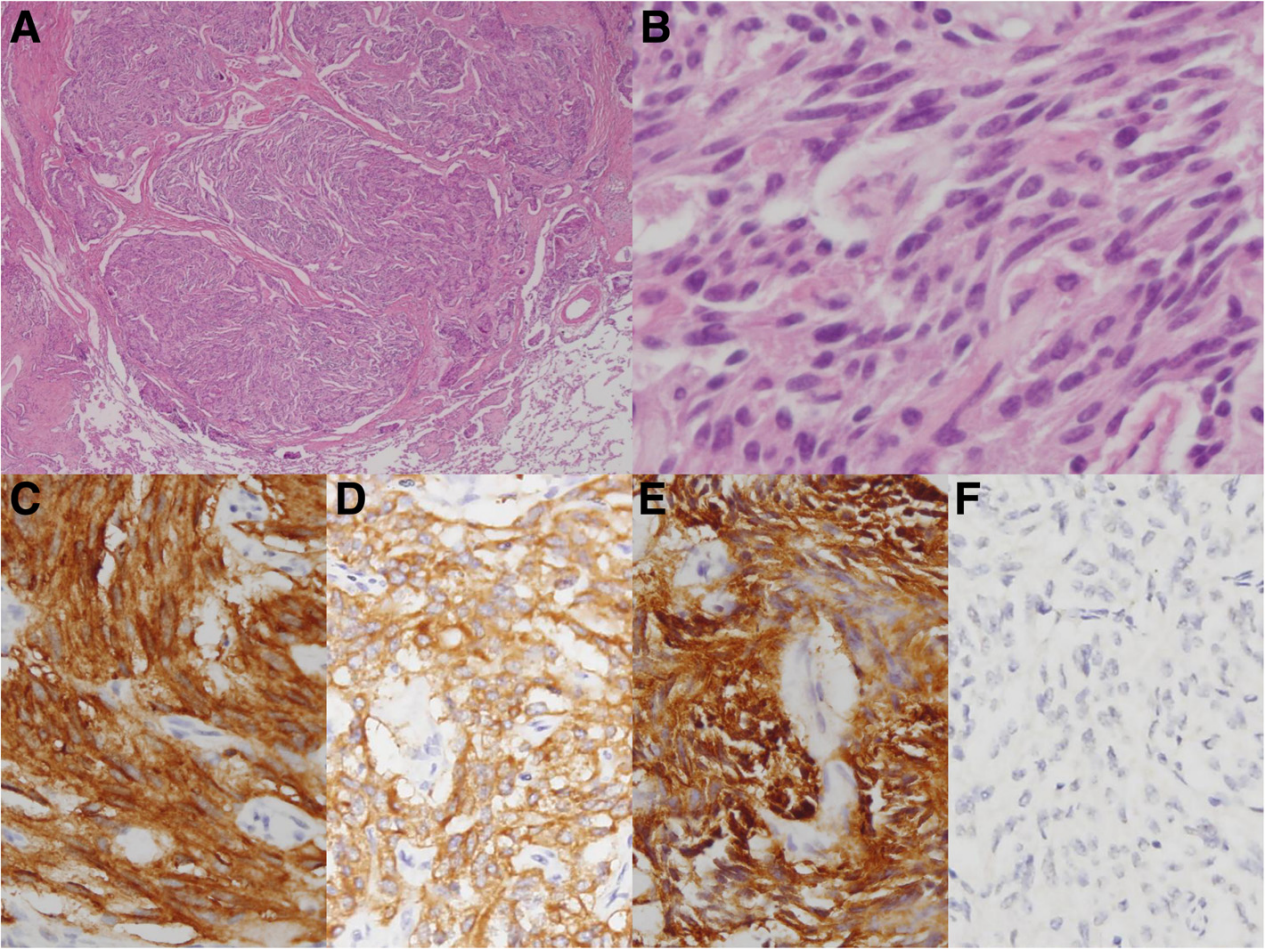
**Postoperative microscopical findings of lesion in the carcinoid portion.** (**A**) The tumor showed trabecular arrangement. (**B**) The tumor composed of monotonous small ovoid to spindle cells with “salt-and pepper” chromatin pattern without mitosis. The tumor cells were positive for CD56 (**C**), synaptophysin (**D**), chromogranin (**E**), but negative for TTF-1 (**F**). (**A**. Original magnification of H&E staining at 20 X; **B**. Original magnification of H&E staining at 400 X; **C-F**. Original magnification of immunohistochemical staining at 200 X).

**Figure 4:**
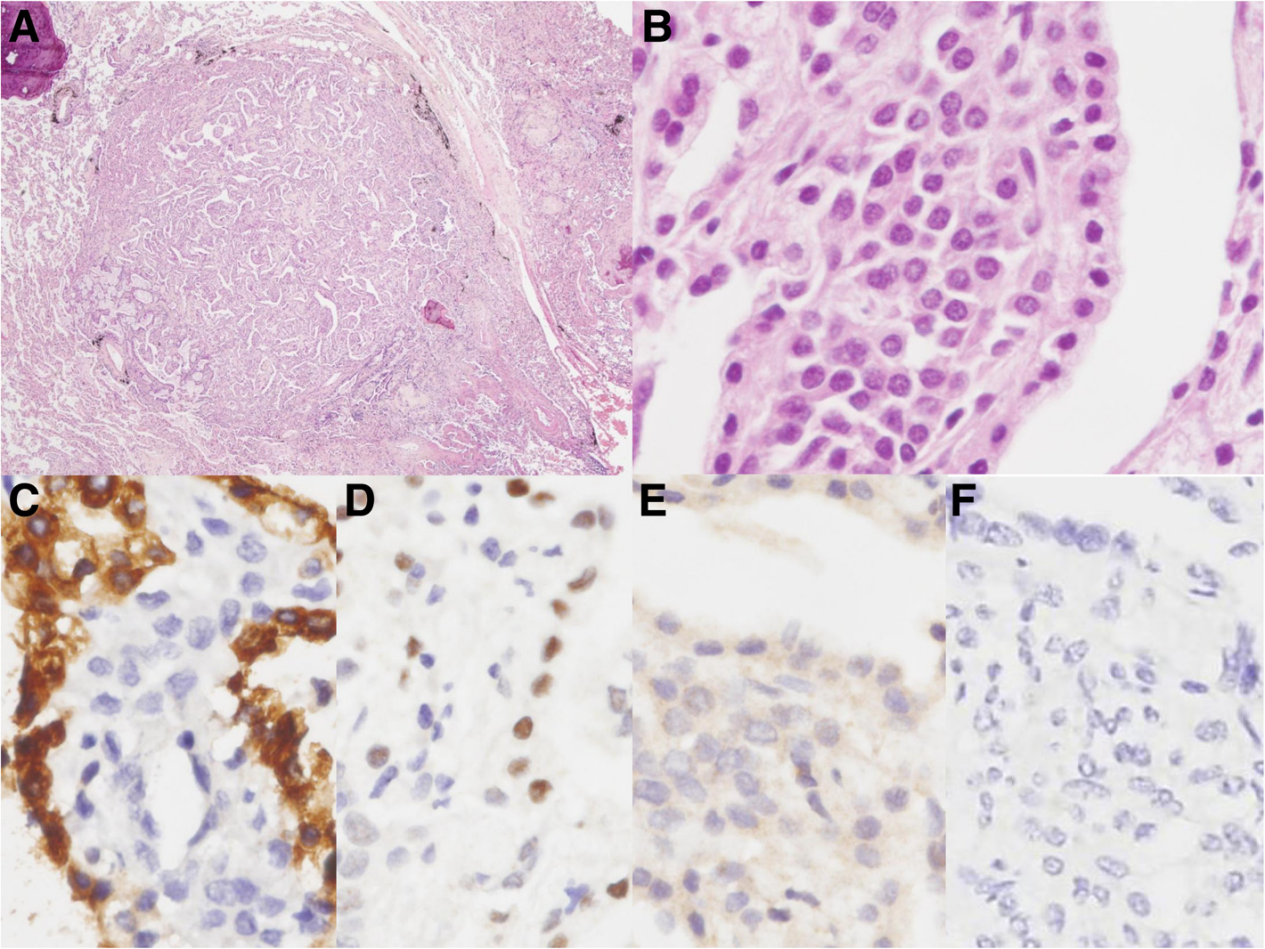
**Postoperative microscopical findings of lesion in the PSH portion.** (**A**, **B**) The tumor shows papillary structures which are composed of hyalinized stalks lined with surface cuboidal cells which shows mild atypical nuclei with vacuolated and foamy cytoplasm. The sclerosing lesion contained round cells with uniform nuclei with pale, eosinophilic or clear cytoplasm. (**C**) On immunohistochemical stain, surface cuboidal cells were positive for pancytokeratin but round cells were negative for it. Both surface cuboidal cells and round cells were positive for TTF-1 (**D**) and negative for synaptophysin (**E**) and chromogranin (**F**). (**A**. Original magnification of H&E staining at 20 X; **B**. Original magnification of H&E staining at 400 X; **C-F**. Original magnification of immunohistochemical staining at 400 X).

Microscopically, nodules were comprised of monotonous small ovoid to spindle cells with “salt-and pepper” chromatin pattern without necrosis and mitosis (Figure 3A-B). The tumor cells were positive for pancytokeratin (CK) and strongly positive for neuroendocrine markers; CD56 (Figure 3C), synaptophysin (Syn) (Figure 3D) and chromogranin (CG) (Figure 3E), but were negative for thyroid transcription factor-1 (TTF-1) (Figure 3F). These demonstrate typical morphologic features of carcinoid tumor (Figure 3A-F). There were no proliferating pulmonary neuroendocrine cells confined within the bronchial and bronchiolar epithelium, which indicates that there was no evidence of diffuse idiopathic pulmonary neuroendocrine cell hyperplasia (DIPNECH). The other nodules in the lung parenchyma showed two different histologic types. Some nodules showed solid and sclerotic patterns composed of surface cuboidal cells and sheets of round cells forming small tubule like architectures (Figure 4A-B). The surface cuboidal cells had mild nuclear atypia with vacuolated foamy cytoplasm. The round cells showed uniform medium-sized polygonal nuclei with slightly pale eosinophilic or clear cytoplasm. Other nodules showed papillary structures which were composed of hyalinized stalks lined by surface cuboidal cells. Necrosis or mitotic figures were not observed (Figure 4B). On immunohistochemical staining, the surface cuboidal cells were positive for CK, epithelial membrane antigen (EMA), and TTF-1, but the round cells were positive for EMA, TTF-1 and negative for CK (Figure 4C-D). Both types of cells were negative for these neuroendocrine markers: Syn (Figure 4E), CG (Figure 4F) and CD56. This morphology represents classical features of PSH (Figure 4A-F). The distribution pattern of these nodules was random and the carcinoid tumors and PSHs were completely separate from each other. Generally, the nodules of carcinoid tumors were larger than the nodules of the PSH. Other pulmonary parenchymal diseases were not seen.

Integrating all the findings from above, the pathological diagnosis of this case was made as multiple peripheral typical carcinoid tumors associated with sclerosing hemangiomas in the lung. The patient did not receive any further treatments post-operation. The size of the residual nodules in the right middle lobe remains unchanged and no new lesion or recurrence has been detected for past 3 years of follow-up period.

## Discussion

Carcinoid tumor is a malignant neuroendocrine neoplasm that rarely presents in the lungs. Such rare pulmonary carcinoids presenting as multiple carcinoids in a lung is an extremely unusual case [7]. Pulmonary carcinoid tumor cells are believed to originate from Kulchistsky cells, located in the bronchial epithelial layer. Multiple carcinoid tumors are thought to be associated with diffuse neuroendocrine cell hyperplasia or carcinoid tumorlets that may cause small airway obstruction [8]. However, there are also some cases that report about multiple peripheral carcinoid tumors or tumorlets without underlying neuroendocrine cell hyperplasia [9]. In this patient’s specimen, there were no findings of diffuse neuroendocrine cell hyperplasia or carcinoid tumorlets. These findings suggest that the neuroendocrine cell hyperplasia is not an obligate preneoplastic lesion of multiple carcinoids. The underlying mechanism of multiple tumors developing synchronously in the lung remains uncertain. One study reports a case about multiple carcinoid tumors or tumorlets in the lung. Patients in this study had a past history of various malignancies such as breast cancer, endometrial adenocarcinoma, colon adenocarcinoma, thyroid papillary carcinoma and malignant melanoma [9]. Primary carcinoid tumors found in combination with different types of tumors in other organs, such as in kidney or colon, have been reported in several studies [10-13]. However, a case about carcinoid tumors found in combination with different types of tumor within a lung has never been reported. The prognoses of multiple carcinoid tumors are unpredictable. Kayser et al. describes that typical carcinoid tumors without lymph node metastasis show a good prognosis [14].

In this case study, synchronous tumors found together with the carcinoid tumors were PSH. Majority of PSH occur as solitary lesion [15-17]. Occasionally they can present as multiple lesions and several studies have reported such cases [18-23]. One paper describes a case of unilateral multiple PSH, where large nodules measuring up to 3 cm surrounded with small nodules measuring from less than 1 cm up to 2.5 cm were observed [18]. Moreover, few cases of bilateral PSH have been reported [20-23]. All of these unilateral and bilateral multiple PSH cases had no events of recurrence or changes throughout the follow up period of 15 months to 9 years post-surgery.

Although PSH is usually considered as benign, several literatures report about metastasizing PSH [3,24-29]. They describe about metastasis involving single, regional or mediastinal lymph nodes. Distant metastasis from the PSH has not yet been reported but a case of local recurrence of PSH from the previous wedge-resection site 10 years post-surgery has been reported [30]. Clinical behaviors of multiple PSH, whether the tumors have developed multicentrically or have metastasized from the primary tumor, remain unknown.

Cases of PSH coexisting with other tumors or diseases have been reported in other papers. These include PSH presenting in combination with: atypical alveolar hyperplasia (AAH), alveolar adenoma, primary pulmonary adenocarcinoma, metastatic hereditary non-polyposis colorectal cancer, and primary adenocarcinoma within the same lobe of the lung [3-6,31]. The authors hypothesized that the AAH or adenocarcinoma-like lesion may be a precursor lesion leading to PSH. There have been two cases, where a carcinoid tumorlet was found within the PSH. Additional two cases report about multiple carcinoid tumorlets ranging in size from 1 mm up to 2.5 mm found adjacent to PSH [16,18]. In this current study, multiple PSHs combined with considerably larger carcinoid tumors, sizes ranging from 5 mm up to 26 mm were found. These two types of tumors presented as separate lesions and the distribution pattern was random. The underlying relationship between the PSH and co-existing disease remains uncertain. It is difficult to clarify the characteristics of PSH due to its rarity and various biological behaviors.

Some studies describe that the round cells and the surface cells in PSH express different immunoreactivity. Both the round cells and the surface cells are positive for TTF-1 and EMA. The surface cells are positive for CK and negative for ER, PR, and Syp while the round cells are negative for CK and can be positive for ER, PR, and Syp. In this current study, the round cells of the PSH showed immuno-positivity for TTF-1 and were negative for Syp. Literature suggests that the positivity of TTF-1 and Syp indicates cells originating from the primitive respiratory cells [6]. Syp is usually positive in neuroendocrine tumors such as in carcinoid tumors. Thus, the author suggests hypothesis that the presence of Syp in the PSH may be involved in the development of typical carcinoid arising from the PSH [20].

## Conclusion

There are few reports about PSH presenting as a single lesion with adjacent carcinoid tumorlets. However, a case of multiple carcinoid tumors presenting together with multiple PSHs have never been reported in the literature. This paper is the first case report of multiple typical carcinoid tumors associated with multiple sclerosing hemangiomas of the lung.

## Consent

Written informed consent was obtained from the patient for publication of this Case Report and any accompanying images. A copy of the written consent is available for review by the Editor-in-Chief of this journal.

## Competing interest

The authors declare that they have no competing interest.

## Authors’ contributions

YK participated in the design of the study, the sequence alignment, and drafted the manuscript. BJK assisted in drafting the manuscript. IJO and SYS made contributions to acquisition of clinical data. JHN and CSP made contributions for analysing the histological features of this case by H&E staining and immunohistochemical staining. YDC revised manuscript critically for important intellectual content. All authors read and approved the final manuscript.

## References

[B1] Liebow AA Hubbell DS Sclerosing hemangioma (histiocytoma, xanthoma) of the lung Cancer 1956 9 53 75 10.1002/1097-0142(195601/02)9:1<53::AID-CNCR2820090104>3.0.CO;2-U 13284701

[B2] Ttravis WD Brambilla W Muller-Hermelink HK Harris CC World health organization classification of tumors: pathology and genetics of tumors of the lung, pleura, thymus and heart 2004 ICRC Press

[B3] Nicholson AG Magkou C Snead D Vohra HA Sheppard MN Goldstraw P Beddow E Hansell DM Travis WD Corrin B Unusual sclerosing haemangiomas and sclerosing haemangioma-like lesions, and the value of TTF-1 in making the diagnosis Histopathology 2002 41 404 413 10.1046/j.1365-2559.2002.01522.x 12405908

[B4] Suzuki K Shiono S Kato H Yanagawa N Sato T [Small sclerosing hemangioma combined with primary lung cancer; report of a case] Kyobu Geka 2006 59 590 593 16856537

[B5] Schiergens TS Khalil PN Mayr D Thasler WE Angele MK Hatz RA Jauch KW Kleespies A Pulmonary sclerosing hemangioma in a 21-year-old male with metastatic hereditary non-polyposis colorectal cancer: report of a case World J Surg Oncol 2011 9 62 10.1186/1477-7819-9-62 21645337 PMC3118379

[B6] Liu W Tian XY Li Y Zhao Y Li B Li Z Coexistence of pulmonary sclerosing hemangioma and primary adenocarcinoma in the same nodule of lung Diagn Pathol 2011 6 41 10.1186/1746-1596-6-41 21599956 PMC3117760

[B7] Yazici U Gulhan E Agackiran Y Tastepe I Yaran P Synchronous bilateral multiple typical pulmonary carcinoid tumors Ann Thorac Surg 2010 89 1278 1280 10.1016/j.athoracsur.2009.09.003 20338356

[B8] Jang BG Kim SY Park SH Multiple pulmonary atypical carcinoids presenting with long-standing cushing syndrome masked by pulmonary tuberculosis Pathol Int 2009 59 399 404 10.1111/j.1440-1827.2009.02384.x 19490471

[B9] Aubry MC Thomas CF Jr Jett JR Swensen SJ Myers JL Significance of multiple carcinoid tumors and tumorlets in surgical lung specimens: analysis of 28 patients Chest 2007 131 1635 1643 10.1378/chest.06-2788 17400673

[B10] Aslam MI Salha IB Muller S Jameson JS Synchronous ileal carcinoid and primary colonic neoplasms: a case report Cases J 2009 2 8317 10.4076/1757-1626-2-8317 19918418 PMC2769428

[B11] Armah HB Parwani AV Primary carcinoid tumor arising within mature teratoma of the kidney: report of a rare entity and review of the literature Diagn Pathol 2007 2 15 10.1186/1746-1596-2-15 17509135 PMC1884130

[B12] Armah HB Parwani AV Perepletchikov AM Synchronous primary carcinoid tumor and primary adenocarcinoma arising within mature cystic teratoma of horseshoe kidney: a unique case report and review of the literature Diagn Pathol 2009 4 17 10.1186/1746-1596-4-17 19523243 PMC2704177

[B13] Cioffi U De Simone M Ferrero S Ciulla MM Lemos A Avesani EC Synchronous adenocarcinoma and carcinoid tumor of the terminal ileum in a Crohn’s disease patient BMC Cancer 2005 5 157 10.1186/1471-2407-5-157 16336666 PMC1322224

[B14] Kayser K Kayser C Rahn W Bovin NV Gabius HJ Carcinoid tumors of the lung: immuno- and ligandohistochemistry, analysis of integrated optical density, syntactic structure analysis, clinical data, and prognosis of patients treated surgically J Surg Oncol 1996 63 99 106 10.1002/(SICI)1096-9098(199610)63:2<99::AID-JSO6>3.0.CO;2-J 8888802

[B15] Katzenstein AL Gmelich JT Carrington CB Sclerosing hemangioma of the lung: a clinicopathologic study of 51 cases Am J Surg Pathol 1980 4 343 356 10.1097/00000478-198008000-00003 6252791

[B16] Rodriguez-Soto J Colby TV Rouse RV A critical examination of the immunophenotype of pulmonary sclerosing hemangioma Am J Surg Pathol 2000 24 442 450 10.1097/00000478-200003000-00014 10716159

[B17] Kuo KT Hsu WH Wu YC Huang MH Li WY Sclerosing hemangioma of the lung: an analysis of 44 cases J Chin Med Assoc 2003 66 33 38 12728972

[B18] Devouassoux-Shisheboran M Hayashi T Linnoila RI Koss MN Travis WD A clinicopathologic study of 100 cases of pulmonary sclerosing hemangioma with immunohistochemical studies: TTF-1 is expressed in both round and surface cells, suggesting an origin from primitive respiratory epithelium Am J Surg Pathol 2000 24 906 916 10.1097/00000478-200007000-00002 10895813

[B19] Niho S Suzuki K Yokose T Kodama T Nishiwaki Y Esumi H Monoclonality of both pale cells and cuboidal cells of sclerosing hemangioma of the lung Am J Pathol 1998 152 1065 1069 9546367 PMC1858231

[B20] Lee ST Lee YC Hsu CY Lin CC Bilateral multiple sclerosing hemangiomas of the lung Chest 1992 101 572 573 10.1378/chest.101.2.572 1310458

[B21] Soumil VJ Navin B Sangeeta D Na J Sharma S Deshpande R Multiple sclerosing hemangiomas of the lung Asian Cardiovasc Thorac Ann 2004 12 357 359 10.1177/021849230401200416 15585708

[B22] Hanaoka J Ohuchi M Inoue S Sawai S Tezuka N Fujino S Bilateral multiple pulmonary sclerosing hemangioma Jpn J Thorac Cardiovasc Surg 2005 53 157 161 10.1007/s11748-005-0024-8 15828298

[B23] Maeda R Isowa N Miura H Tokuyasu H Kawasaki Y Yamamoto K Bilateral multiple sclerosing hemangiomas of the lung Gen Thorac Cardiovasc Surg 2009 57 667 670 10.1007/s11748-009-0452-y 20013104

[B24] Chan AC Chan JK Pulmonary sclerosing hemangioma consistently expresses thyroid transcription factor-1 (TTF-1): a new clue to its histogenesis Am J Surg Pathol 2000 24 1531 1536 10.1097/00000478-200011000-00009 11075855

[B25] Tanaka I Inoue M Matsui Y Oritsu S Akiyama O Takemura T Fujiwara M Kodama T Shimosato Y A case of pneumocytoma (so-called sclerosing hemangioma) with lymph node metastasis Jpn J Clin Oncol 1986 16 77 86 3009921

[B26] Yano M Yamakawa Y Kiriyama M Hara M Murase T Sclerosing hemangioma with metastases to multiple nodal stations Ann Thorac Surg 2002 73 981 983 10.1016/S0003-4975(01)03122-8 11899220

[B27] Chan NG Melega DE Inculet RI Shepherd JG Pulmonary sclerosing hemangioma with lymph node metastases Can Respir J 2003 10 391 392 14571291 10.1155/2003/534147

[B28] Miyagawa-Hayashino A Tazelaar HD Langel DJ Colby TV Pulmonary sclerosing hemangioma with lymph node metastases: report of 4 cases Arch Pathol Lab Med 2003 127 321 325 12653576 10.5858/2003-127-0321-PSHWLN

[B29] Kim GY Kim J Choi YS Kim HJ Ahn G Han J Sixteen cases of sclerosing hemangioma of the lung including unusual presentations J Korean Med Sci 2004 19 352 358 10.3346/jkms.2004.19.3.352 15201499 PMC2816834

[B30] Wei S Tian J Song X Chen Y Recurrence of pulmonary sclerosing hemangioma Thorac Cardiovasc Surg 2008 56 120 122 10.1055/s-2007-989280 18278694

[B31] Noguchi M Kodama T Morinaga S Shimosato Y Saito T Tsuboi E Multiple sclerosing hemangiomas of the lung Am J Surg Pathol 1986 10 429 435 10.1097/00000478-198606000-00008 3717498

